# Independent and Parallel Evolution of New Genes by Gene Duplication in Two Origins of C4 Photosynthesis Provides New Insight into the Mechanism of Phloem Loading in C4 Species

**DOI:** 10.1093/molbev/msw057

**Published:** 2016-03-24

**Authors:** David M. Emms, Sarah Covshoff, Julian M. Hibberd, Steven Kelly

**Affiliations:** ^1^Department of Plant Sciences, University of Oxford, Oxford, United Kingdom; ^2^Department of Plant Sciences, University of Cambridge, Cambridge, United Kingdom

## Abstract

C_4_ photosynthesis is considered one of the most remarkable examples of evolutionary convergence in eukaryotes. However, it is unknown whether the evolution of C_4_ photosynthesis required the evolution of new genes. Genome-wide gene-tree species-tree reconciliation of seven monocot species that span two origins of C_4_ photosynthesis revealed that there was significant parallelism in the duplication and retention of genes coincident with the evolution of C_4_ photosynthesis in these lineages. Specifically, 21 orthologous genes were duplicated and retained independently in parallel at both C_4_ origins. Analysis of this gene cohort revealed that the set of parallel duplicated and retained genes is enriched for genes that are preferentially expressed in bundle sheath cells, the cell type in which photosynthesis was activated during C_4_ evolution. Furthermore, functional analysis of the cohort of parallel duplicated genes identified SWEET-13 as a potential key transporter in the evolution of C_4_ photosynthesis in grasses, and provides new insight into the mechanism of phloem loading in these C_4_ species.

Key words: C4 photosynthesis, gene duplication, gene families, parallel evolution.

## Introduction

The evolution of C_4_ photosynthesis from ancestral C_3_ photosynthesis involved multiple changes to biochemistry, subcellular compartmentalization, and leaf anatomy such that most modern-day C_4_ species partition the entire photosynthetic process between two discrete cell types—bundle sheath (BS) and mesophyll (M) cells. This partitioning of the photosynthesis reactions between two cells facilitates an increase in CO_2_ concentration around Rubisco leading to a reduction in photorespiration and a concomitant increase in photosynthetic efficiency (Hatch et al. 1967). Despite the numerous differences between C_3_ and C_4_ species, C_4_ photosynthesis has evolved independently in over 60 different plant lineages (Sage et al. 2011) and thus represents one of the most widespread and successful examples of convergent evolution in eukaryotic biology.

Several complementary studies have identified patterns of gene expression associated with the evolution of C_4_ photosynthesis. Comparisons of gene expression between closely related C_3_ and C_4_ species in the genera *Cleome* and *Flaveria* have identified ∼3,500 gene expression changes associated with the evolutionary transition from C_3_ to C_4_ with smaller subsets of genes changing in parallel in multiple lineages (Gowik et al. 2011). Other studies identified genes with expression profiles that correlate with biochemical (Li et al. 2010; Majeran et al. 2010; Pick et al. 2011; Sage and Zhu 2011) or anatomical (Wang et al. 2013) features of C_4_ species, or with biochemical differences between specialized C_4_ cell types (Li et al. 2010; Chang et al. 2012; John et al. 2014). Further studies have also identified parallel recruitment of transfactors in two distantly related origins of C_4_ (Aubry et al. 2014) and parallel recruitment of *cis*-elements to control C_4_ gene expression (Brown et al. 2011). Although our understanding of gene expression changes associated with C_4_ biology is rapidly increasing, almost nothing is known about the gene content differences that distinguish C_4_ species from their C_3_ relatives.

Individual gene and whole-genome duplications are a dominant feature of plant evolution and have been detected in all angiosperm lineages (Adams and Wendel 2005; Soltis et al. 2009). Although gene duplication is a stochastic process, retention of duplicate genes is affected by selection, and duplicates are frequently lost (Lynch and Conery 2000). In the case of whole-genome duplication events this loss process is known as fractionation (Thomas et al. 2006; Woodhouse et al. 2010). If retained, the duplicated genes may share the original gene function, they may partition the original gene function (subfunctionalization), or they may diverge and develop novel functions (neofunctionalization) (Ohno 1970; Lynch and Conery 2000; Moore and Purugganan 2005; Freeling et al. 2008). In maize, duplicated genes that share the same expression pattern evolve faster than those whose expression profiles diverge in time or space, and are more likely to be subject to positive selection (Hughes et al. 2014).

Although gene duplication has been studied in specific genes or at a genome scale in individual species such as *Arabidopsis thaliana*, *Glycine max*, and *Zea mays* (Blanc and Wolfe 2004; Duarte et al. 2006; Erdmann et al. 2010; Liu et al. 2011; Guo et al. 2013; Roulin et al. 2013; Hughes et al. 2014), it is unknown to what extent gene duplication events occur in parallel across species and to what extent these events underlie the emergence of complex convergent phenotypes. It is also unknown whether the evolution of C_4_, and the changes in photosynthetic competence of the bundle sheath and mesophyll cells, is associated with gene duplication. Analysis of gene expression data from different C_4_ species has led some authors to propose that neofunctionalization of genes following duplication has not played a major role in the evolution of C_4_ photosynthesis (Gowik and Westhoff 2011; Williams et al. 2012; van den Bergh et al. 2014). However, these analyses have primarily focused on the limited number of genes already known to be involved in C_4_ photosynthesis, and have not taken ab initio approaches to the discovery of duplicated genes associated with the evolution of C_4_ photosynthesis. Although gene duplication events associated with the evolution of C_4_ have yet to be identified, several lines of evidence show that gene duplications that predate the origin of C_4_ photosynthesis have played an important role in facilitating its emergence (Monson 1999, 2003; Sage 2004). Archetypal examples of this include specific members of the PEPC (phosphoenolpyruvate carboxylase), CA (carbonic anyhdrase), and other gene families that are repeatedly co-opted for use in the C_4_ cycle in multiple independent C_4_ origins (Aldous et al. 2014; Christin et al. 2015).

Here we exploit recent advances in the ability to infer gene families and accurately localize gene duplication events (Boussau et al. 2013; Emms and Kelly 2015) to perform a genome-wide gene-tree species-tree reconciliation of all genes from seven monocot species that span two origins of C_4_ photosynthesis. We reveal that many C_4_ cycle genes duplicated in the common ancestor of the sampled *Poaceae* species, prior to the evolution of C_4_. We show that subsequent co-option for C_4_ function of particular genes from these duplicate pairs was likely a result of expression divergence prior to the diversification of the *Poaceae*. We also provide an analysis of gene duplication associated with the evolution of C_4_ photosynthesis and reveal that there is significant independent, parallel duplication and subsequent retention of genes concurrent with the evolution of C_4_ photosynthesis. Furthermore, we show that genes that duplicated independently at both C_4_ origins are preferentially expressed in BS cells and comprise a suite of genes with novel functional significance for the evolution of C_4_ photosynthesis.

## New Approaches

Here we take a novel approach to elucidating the evolutionary changes that occurred in the transition from C_3_ to C_4_ photosynthesis in two independent lineages of C_4_ species. Through genome-wide gene-tree species-tree reconciliation, we reveal that hundreds of new genes evolved by gene duplication coincident with the evolution of C_4_ photosynthesis in both lineages. Furthermore, comparative analysis of these gene duplications reveals that there is commonality in the duplicated genes with a core set of 21 genes that duplicated and were retained independently in parallel in both lineages.

## Results

### Inference of Orthogroups and Identification of Gene Duplication Events

**Fig. 1:**
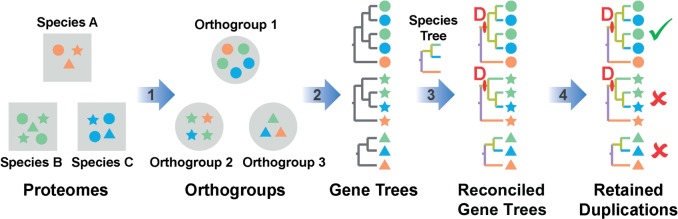
Schematic overview of the gene duplication identification method used in this analysis. 1) Clustering genes into orthogroups using OrthoFinder. 2) Construction of multiple sequence alignments using MAFFT and inference of gene trees using Phyldog. 3) Reconciliation of gene trees with species tree using Phyldog. 4) Filtering of reconciled gene trees to identify gene duplication events that are retained in all species that evolved following the gene duplication event.

**Fig. 2:**
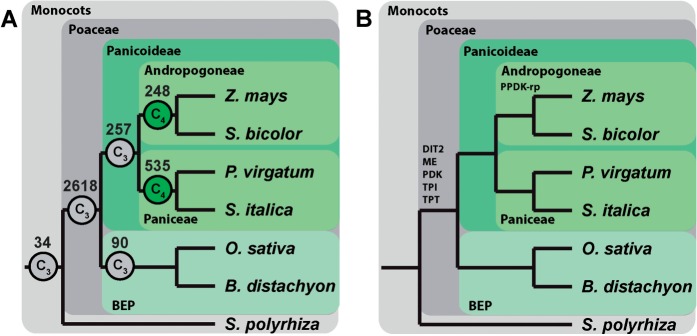
(*A*) The number of fully retained gene duplication events that occurred along each nonterminal branch during the evolution of the monocot species in the study. The branches on which C _4_ evolved are indicated by green circles. (*B*) The C_4_ cycle associated genes that duplicated during this same period. Genes are depicted above the branch on which they duplicated. DIT2, dicarboxylate transporter 2; ME, NADP-dependent malic enzyme; PDK, pyruvate dehydrogenase kinase; TPI, triosephosphate isomerase; TPT, triosephosphate/phosphate translocator; PPDK-rp, pyruvate orthophosphate dikinase regulatory protein.

To provide the data for this analysis, a set of gene families (orthogroups) covering 42 sequenced plant genomes available in Phytozome V10 (Goodstein et al. 2012) was constructed (supplementary file S1 and fig. 1*A*, Supplementary Material online). Those orthogroups that contained sequences from monocot genomes were selected for further analysis (fig. 1). The set of monocots in this analysis comprise two independent origins of C_4_ photosynthesis (fig. 2*A*) (Aliscioni et al. 2012), an origin in the *Andropogoneae* of which *Z.* *mays* and *Sorghum* *bicolor* are descendants and an independent origin in the *Paniceae* of which *Setaria* *italica* and *Panicum virgatum (DOE-JGI)* are descendants. Although biochemical analyses suggested that *P.* *virgatum* and *Se.* *italica* were descendants of independent C_4_ origins (Hattersley and Watson 1992), the most recent phylogenetic analysis suggests that they share a single origin (Aliscioni et al. 2012). The independence of the C_4_ origins in this study is supported by the presence of a number of intervening clades containing C_3_ species including the *Arthropogoninae*, *Otachyriinae*, *Boivinellinae*, and *Neurachninae* (Aliscioni et al. 2012). The data set comprised 13,773 orthogroups and contained 248,266 genes (77.5% of all monocot genes) distributed across the 7 sampled monocot species. The phylogenetic position of individual gene duplication events contained within the 13,773 orthogroups were identified through construction of multiple sequence alignments, phylogenetic tree inference, and gene-tree species-tree reconciliation (fig. 1; see Methods). This resulted in the identification of 91,815 gene duplication events within the monocot phylogenetic tree. To identify retained gene duplication events the following two filtration criteria were applied: 1) A gene duplication event must have supporting evidence for its occurrence from at least two species and 2) all genes resulting from a gene duplication event must be retained in the genomes of all species that diverged after the duplication event. In total, 70,101 of the gene duplication events were on terminal branches of the species tree and so did not satisfy the first criterion. Of the remaining 21,714, a total of 3,782 gene duplications (distributed across the monocot phylogeny) satisfied the second criterion (fig. 2*A*), that is, that the genes descended from the gene duplication event were retained in all of the species that diverged after the gene duplication event. The complete set of phylogenetic trees for all these gene duplication events is provided in supplementary file S1, Supplementary Material online, and is available from the Dryad Digital Repository, http://dx.doi.org/10.5061/dryad.17m42. A Gene Ontology (GO) term enrichment analysis of the orthogroups in which these duplications occurred is provided in supplementary file S2, Supplementary Material online.

### Gene Duplication Events Occurred in Multiple Genes Associated with the C_4_ Cycle

**Fig. 3:**
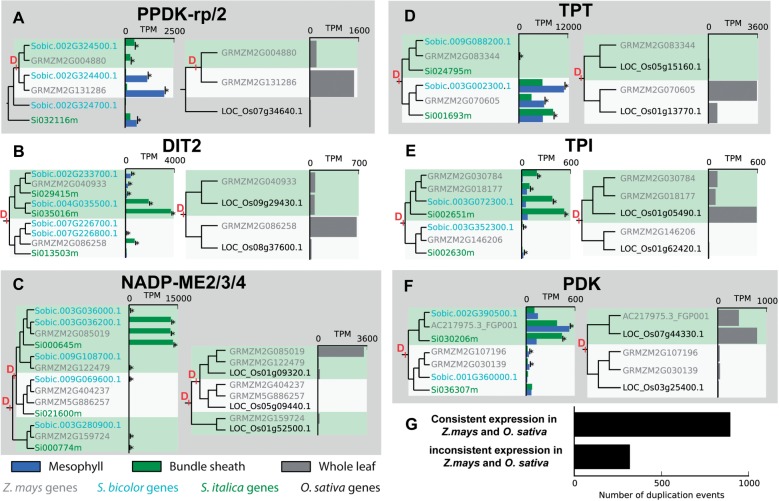
Expression profile of duplicate genes in bundle sheath and mesophyll cells of C_4_ species and in fully expanded foliar leaves of *Zea mays* and *Oryza sativa*. Only genes from species for which the corresponding data are available (BS/M and whole leaf expression) are shown in each tree. The complete trees including *Panicum virgatum* are included in supplementary file S1, Supplementary Material online, and are available from the Dryad Digital Repository, http://dx.doi.org/10.5061/dryad.17m42. Gene duplication events meeting our selection criteria are marked with a “D.” Green and gray backgrounds distinguish genes descended from the two duplicates following the gene duplication event. Green bars indicate expression level in bundle sheath cells, blue bars indicate expression level in mesophyll cells. Gray bays indicate expression level in fully expanded leaves of *Z. mays* and *O. sativa*. TPM, transcripts per million. For explanation of gene abbreviations see legend to figure 2. PPDK-rp/2 duplicated in the ancestor of the *Andropogoneae*, the remainder duplicated in the ancestor of the *Poaceae*. (*G*) The number of duplication events in the ancestor of the *Poaceae* for which the most highly expressed of the duplicate genes in *Z. mays* is consistent (the same duplicate) or inconsistent (the other duplicate) with the most highly expressed of the duplicate genes in *O. sativa*. All duplication events for which one of the descendant genes in *O. sativa* and one of the descendant genes in *Z. mays* are both expressed at greater than 10 transcripts per million are included. For explanation of gene name abbreviations please see the legend to figure 2.

The filtered set of gene duplication events within the monocots contained six genes already known to be associated with the C_4_ cycle (fig. 2*B* and supplementary fig. 1*B*, Supplementary Material online) (Langdale 2011). These comprise PPDK-rp, DIT2, NADP-ME, TPT, TPI, and a gene recently identified as upregulated in multiple C_4_ lineages, PDK (Brautigam et al. 2011, 2014) (fig. 3*A*–*F*). Of these, only pyruvate, orthophosphate dikinase regulatory protein (PPDK-rp) duplicated concurrently with an origin of C_4_ photosynthesis, in the ancestor of *So. bicolor* and *Z. mays* (figs. 2*B* and 3*A*). The remaining five duplicated in the ancestor of the *Poaceae* and three of these—NADP-ME2/3/4, PDK and DIT2—were duplicated in syntenic blocks associated with the ρ whole-genome duplication (Salse et al. 2008; Tang et al. 2010; Lee et al. 2013) while none showed evidence of being duplicated as part of the earlier σ or τ whole-genome duplications that also occurred on this branch of the species tree (Tang et al. 2010; Jiao et al. 2014). Comparison of the expression profile of the PPDK-rp duplicates in *So. bicolor* and *Z. mays* with that of the single copy gene in *Se. italica* shows that subfunctionalization of this gene has occurred in the lineage leading to *So. bicolor* and *Z. mays* (fig. 3*A*). Here, the single PPDK-rp gene in *Se. italica* is expressed in both BS and M cells, while in *So. bicolor* and *Z. mays* one of the duplicate pair is expressed in BS cells and the other is expressed in M cells (fig. 3*A*). All of the duplicate genes as well as the orthologous genes in *Se. italica*, *Oryza* *sativa*, and *Brachypodium* *distachyon* are predicted to encode chloroplast transit peptides. Although these duplicated genes now reside on different chromosomes in the fractionated *Z. mays* genome, they occur as a tandem pair in the genome of *So. bicolor* suggesting that they evolved by tandem duplication prior to the divergence of *So. bicolor* and *Z. mays*.

### Expression Divergence of Duplicate Genes Prior to C_4_ Evolution Determined Recruitment to C_4_ Function

A similar phenomenon is observed for the duplicates of glutamate/oxaloacetate malate exchange transporter, DIT2. Here two gene duplication events occurred, one prior to the divergence of the grasses and a second (not fully retained and hence not included in totals in fig. 2*A*) at the base of the *Panicoideae* (fig. 3*B*)*.* In each species, one of the duplicated genes is expressed in M cells and another in BS cells (fig. 3*B*). The duplicate gene that is expressed in the BS in *So. bicolor* (Sobic.004G035500.1) and *Se. italica* (Si03501m) was lost from *Z. mays* and appears to be compensated for by BS-specific expression of the earlier duplicate (GRMZM2G086258) that originated at the base of the grasses (fig. 3*B*). The most parsimonious explanation for this phenomenon is that the gene used in C_4_ photosynthesis in *Z. mays* today was not the gene utilized for this function when C_4_ first evolved in this lineage. Furthermore, depending on the ancestral expression pattern of the single copy gene in the last common ancestor of the *Panicoideae*, the observed, divergent expression profile of the duplicate genes requires that either subfunctionalization or neofunctionalization of these duplicate genes occurred coincident with the evolution of C_4_ photosynthesis (fig. 3*B*).

For the remainder of the gene duplication events in C_4_ cycle genes that occurred prior to the divergence of the grasses (NADP-ME, TPT, TPI, and PDK), the same duplicate of the gene pair generated by the duplication event is expressed more highly in fully expanded leaves of both *O. sativa* (Covshoff et al. 2015) and *Z. mays* (Wang et al. 2013) (fig. 3C–*F*). These gene duplication events that occurred in the ancestor of the grasses ≥50 Ma (Vicentini et al. 2008) predate the origin of C_4_ photosynthesis in the *Andropogoneae* and *Paniceae* by ≥29 My (Christin 2008). The fact that in each case the same duplicate gene was recruited for use in the C_4_ cycle in both independent origins raises the question as to whether in each case gene expression or biochemical changes modified the genes such that one duplicate was better suited for function in C_4_ photosynthesis. Comparison of the mRNA abundance of these duplicated genes in *Z. mays* and *O. sativa* revealed that in all cases the duplicated gene that was recruited for function in C_4_ photosynthesis was the ortholog of the one most highly expressed in mature rice leaves (fig. 3B–*F*) indicating that the duplicated genes had already undergone regulatory neofunctionalization prior to the divergence of the grass species in this study. To determine whether this pattern of duplicate gene recruitment was unique to C_4_ cycle genes, the mRNA abundance of all genes descended from gene duplication events that occurred prior to the divergence of the grasses were analyzed in mature leaves of *Z. mays* and *O. sativa*. In the cases where both duplicate genes were expressed in fully expanded foliar leaves of both species, 74% (*n* = 893/1210) of duplicate pairs exhibited a consistent expression pattern whereby the same gene of the duplicate pair was more highly expressed in the leaves of *Z. mays* and *O. sativa* (fig. 3G). Thus this recruitment phenomenon is not unique to C_4_ cycle genes, and it is more parsimonious to assume that expression differences, rather than biochemical differences, were the determining factors for recruitment to C_4_ function.

### Multiple Gene Duplication Events Occurred in the Same Gene Families at Both Origins of C _4_

**Fig. 4:**
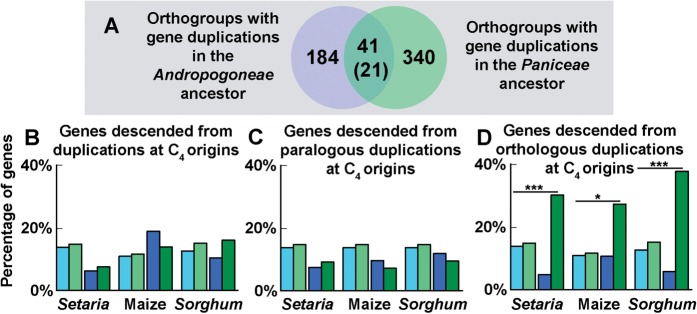
(*A*) Venn diagram showing the overlap between the orthogroups in which gene duplication events occurred. Number in parenthesis is the number of genes in which gene duplications happened to the same gene. (*B*) The expression profile in bundle sheath and mesophyll cells of all genes duplicated at the origins of C_4_ photosynthesis in this study. (*C*) The expression profile in bundle sheath and mesophyll cells of genes descended from paralogous duplication events (duplication in the same orthogroup in both origins). (*D*) The expression profile in bundle sheath and mesophyll cells of genes descended from orthologous duplication events (duplication in the same gene in both origins). Dark blue/green bars show the percentage of genes that are preferentially expressed in M/BS cells. Light blue/green bars show the percentage of all genes in each genome that are preferentially expressed in M/BS cells. ***P <  0.001; *P < 0.05.

The retained gene duplication events that were localized to a branch that contained an origin of C_4_ were compared to determine whether there were any that occurred in parallel in both origins of C_4_. Here a parallel gene duplication event is defined as a gene duplication that occurred independently to the same ortholog in both C_4_ lineages. The 535 gene duplications that occurred in the ancestor of the *Paniceae* and the 248 in the ancestor of the *Andropogoneae* occurred in 381 and 225 orthogroups, respectively (supplementary file S1, Supplementary Material online), as multiple gene duplications occurred in some orthogroups. Of these gene duplications, 41 occurred independently to genes in the same orthogroup at both independent C_4_ origins (fig. 4*A* and supplementary file S3, Supplementary Material online). This overlap is significantly more than would be expected given the number of orthogroups and the number of gene duplication events on each branch (hypergeometric test *P* = 10^−^^21^, expected number = 7). GO term analysis of this overlapping set of orthogroups revealed significant enrichment of multiple functional categories of genes. Of these, ∼24% (10/42) had plausible association with C_4_ photosynthesis, including those categories concerning the biosynthesis of plant cell walls (Edwards and Voznesenskaya 2011), plasmodesmata (Botha 1992; Sowinski et al. 2007), and flavonols (Hannoufa et al. 1994), as well as those concerning leaf morphogenesis and development (Wang et al. 2013) (supplementary file S2, Supplementary Material online).

### Twenty-one Gene Duplication Events Occurred in the Same Genes at Both Origins of C_4_

Of the 41 gene duplication events in the same orthogroup, 21 occurred in parallel whereby the same gene was duplicated at both C_4_ origins and was subsequently retained in all sampled, descendant species (fig. 4*A* and supplementary file S3, Supplementary Material online). These 21 genes comprise two transcription factors, two transporters, two genes involved in hormone metabolism, two genes for redox homeostasis, six genes of unknown function, as well as one gene in each of the following categories: Cell wall biosynthesis, plastid import, flavonoid biosynthesis, lipid metabolism, plantacyanin, component of the ribosome, and signaling component (supplementary file S3, Supplementary Material online). The phylogenetic trees, BS and M cell expression profile, expression profile in fully expanded leaves of *Z. mays* and *O. sativa*, as well as the expression profile of the *Z. mays* genes during leaf development are shown in supplementary figure 2A–U, Supplementary Material online. The complete set of phylogenetic trees for all of these gene duplication events is provided in supplementary file S1, Supplementary Material online, and is available from the Dryad Digital Repository, http://dx.doi.org/10.5061/dryad.17m42.

The evolution of C_4_ photosynthesis in these lineages involved the activation of photosynthesis in the BS. Given this, the BS and M cell expression of the genes in *So. bicolor*, *Se. italica* (John et al. 2014), and *Z. mays* (Chang et al. 2012) that were duplicated on branches that encompassed C_4_ was analyzed. Here, the proportion of duplicated genes that were preferentially expressed in BS or M cells was compared with the proportion of all genes preferentially expressed in these cell types in the same species. Analysis of these expression data revealed that there was no significant enrichment of BS or M preferentially expressed genes in the complete set of genes that duplicated at either C_4_ origin (fig. 4*B*). Neither was there a significant enrichment of BS or M preferentially expressed genes in the set of duplicate genes where the duplication event occurred in the same orthogroup (excluding those that occurred in the same gene; fig. 4*C*). However, there were significantly more BS preferentially expressed genes than expected in the set of genes that duplicated in parallel at both origins of C_4_ photosynthesis (fig. 4*D*). Of the parallel duplicated genes that show BS expression, ∼45% of the descendant pairs are both preferentially expressed in the BS and ∼50% show evidence of regulatory neofunctionalization where one of the duplicates is preferentially expressed in the BS and the other is either not preferentially expressed in either cell type or is preferentially expressed in M cells (supplementary fig. 2X, Supplementary Material online).

### Parallel Duplicated Genes Provide Insight into the Evolution of Phloem Loading in C_4_

**Fig. 5:**
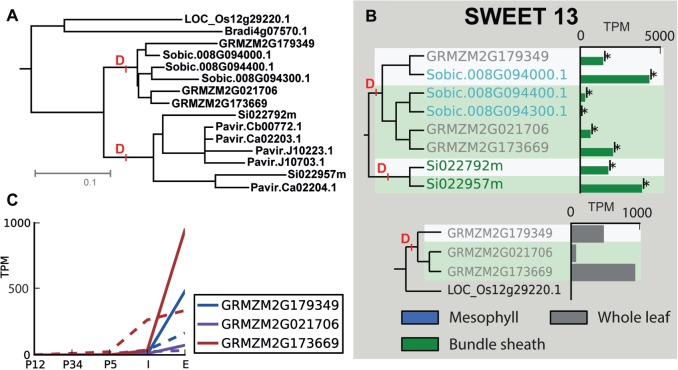
The SWEET-13 sucrose efflux transporter duplicated independently at both C_4_ origins. (*A*) The gene tree showing the independent, parallel gene duplication events. (*B*) The expression profile in bundle sheath (green bars) and mesophyll cells (blue bars) of each duplicate gene and a comparison of the whole leaf expression profile of each duplicate in *Zea mays* to the single copy ortholog in *Oryza sativa*. (*C*) The expression profile of each duplicate gene in *Z. mays* foliar (solid lines) and husk (dashed lines) leaf developmental gradient.

In C_4_ *Panacoid* grasses the plasmodesmata connections between BS and vascular parenchmya are significantly less numerous than those between M and BS (Botha 1992; Botha and Vanbel 1992; Sowinski et al. 2007). In addition, the plasmodesmata connections between the BS and the vascular parenchyma are significantly less numerous than those observed in related C_3_ species (Botha 1992; Botha and Vanbel 1992; Sowinski et al. 2007), and the mechanism by which sucrose is unloaded from BS cells into the vasculature is unknown. One of the two transporters that duplicated in parallel at both origins of C_4_ (fig. 5*A*) is the ortholog of the *O. sativa* SWEET-13 sucrose efflux transporter (Zhou et al. 2015). Although SWEET-13 in *O. sativa* is expressed to a low level in mature leaves (fig. 5*B*), the duplicate genes are highly expressed in whole leaves in *Z. mays* and are each among the most highly expressed genes in the BS (e.g., GRMZM2G173669, GRMZM2G179349, and GRMZM2G021706 are the 57th, 87th, and 166th most highly expressed genes in the BS respectively of 63,480 genes in *Z. mays*). It is possible, given the isolation protocol (Covshoff et al. 2013), that in *Se. italica* and *So. bicolor* some expression of SWEET-13 occurs in the vasculature rather than the BS. However, this is not the case for *Z. mays* where BS transcriptome data comes from mechanically isolated (Chang et al. 2012) and laser dissected BS cells (Li et al. 2010). Moreover, the genes are most highly expressed in fully expanded foliar leaves when compared with other developmental stages (fig. 5*C*).

### The Putative Functional Significance of Other Parallel Duplicated Genes

Of note is that 2 of the 21 parallel duplication events occurred in the same orthogroup of glutaredoxin genes (comprising four duplications in total). In total, 14 of the 17 genes descended from these duplication events are preferentially expressed in BS cells (supplementary fig. 2A and *B*, Supplementary Material online). Moreover, all of the maize genes are expressed more highly in photosynthetically active tissue than in young developing tissue (supplementary fig. 2A and 2*B*, Supplementary Material online). Glutaredoxin genes are involved in antioxidant defense and stimulate photorespiratory glycine decarboxylase activity in plant mitochondria (Hoffmann et al. 2013). Thus given the expression of the duplicate genes in BS cells, it is possible that they function to protect the cell from oxidative damage as a result of activation of photosynthesis.

As described above, many of the parallel duplications occurred in genes of unknown function. Although some of these duplicated genes have clues to function from the presence of conserved protein domains such as the parallel duplication that occurred in the ABC-type transporter (supplementary fig. 2S, Supplementary Material online), or the multiple parallel duplications that occurred in the glutaredoxin gene (above), many have no domains or other features that can provide clues to potential protein function. The most striking example of these duplications in unknown genes is that of the gene duplication events that happened in the orthogroup shown in supplementary figure 2L, Supplementary Material online. Here the majority of genes (14/20) are preferentially expressed in the BS cells of C_4_ species. Moreover, during *Z. mays* leaf development they are also more highly expressed in immature and fully expanded leaves than in earlier developmental stages. Although no clues to function can be obtained from the analysis of the protein sequence, it seems likely that the gene family provides some function to BS cells.

### The Transcription Factors That Duplicated in Parallel at Both Origins of C4

**Fig. 6:**
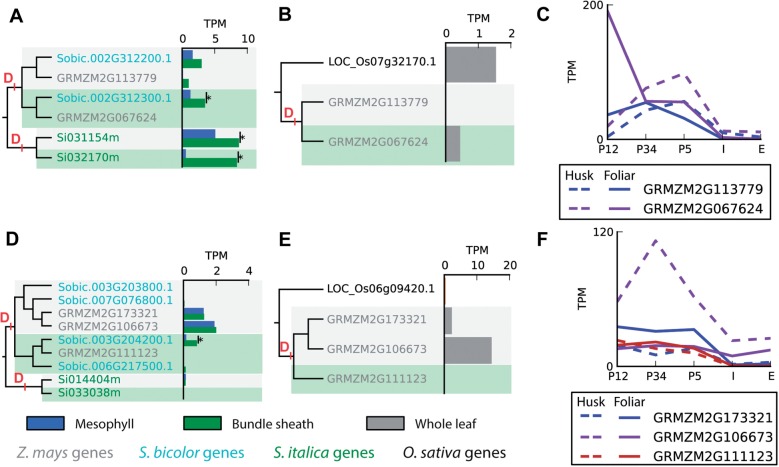
Two transcription factors duplicated independently at both C4 origins. (*A*) SQUAMOSA promoter binding-like transcription factor, *A*–*C*; an AP2/B3 transcription factor, *D*–*F*. (*A* and *D*) The expression profile in bundle sheath (green bars) and mesophyll cells (blue bars) of each duplicate gene. (*B* and *E*) A comparison of the whole leaf expression profile of each duplicate in *Zea mays* to the single copy ortholog in *O. sativa*. (*C* and *F*) The expression profile of each duplicate gene in *Z. mays* foliar (solid lines) and husk (dashed) leaf developmental gradient.

In total, 40 transcription factors (*Andropogoneae* = 15, *Paniceae* = 25) duplicated at the 2 independent C_4_ origins in this study. Thus significant novel regulatory capacity evolved coincident with the evolution of C_4_ photosynthesis in these lineages. Of these 40 gene duplication events, 2 duplicated in parallel at both origins of C_4_ photosynthesis and the descendants of both are most highly expressed during early stages of leaf development (fig. 6C and *F*). One of the transcription factors that duplicated independently at both C_4_ origins is a SQUAMOSA promoter binding-like transcription factor (fig. 6*A*). Although neither the duplicate genes in the C_4_ species nor the related single copy gene in rice have been functionally characterized, other members of the gene family have been shown to modulate plant architecture and development (Jorgensen and Preston 2014). The duplicate genes are primarily expressed in early developmental stages of both foliar and husk leaves in *Z. mays* (fig. 6B and 6*C*). Analysis of the expression profile of the duplicate genes (GRMZM2G113779 and GRMZM2G067624) during leaf development in *Z. mays* shows that the duplicate gene pair has undergone regulatory neofunctionalization (fig. 6*C*). Moreover, one of the duplicates is highly expressed at the initial stages of foliar leaf development (fig. 6*C*) where Kranz anatomy is patterned (Wang et al. 2013). It is noteworthy that even though expression of these genes decreases as leaf tissue matures, in mature leaves the duplicate transcription factors still exhibit preferential expression in BS cells.

The other parallel duplicated transcription factor is an AP2/B3 transcription factor (fig. 6*D* and *F*). These duplicated transcription factor genes are also more highly expressed in developing leaf tissue as compared with fully expanded leaves, and comparison of the expression profile of the duplicate genes during foliar and husk leaf development in maize provides evidence of regulatory neofunctionalization in these leaf types (fig. 6*F*).

## Discussion

The evolution of C_4_ photosynthesis required numerous changes to plant anatomy and biochemistry such that many differences distinguish C_4_ species from their C_3_ relatives. Although a significant body of knowledge has amassed concerning genes that are differentially expressed between C_4_ and C_3_ species, it is unknown whether the evolution of C_4_ photosynthesis was concomitant with the evolution of new genes. Here we have shown that hundreds of new genes evolved by gene duplication coincident with the evolution of C_4_ photosynthesis and were subsequently retained in two independent lineages of C_4_ species, revealing a previously hidden complexity to C_4_ evolution. Although any of these gene duplication events may be important to the evolution of C_4_, many are likely linked to other traits that evolved during the same period captured by the branch in the phylogenetic tree on which C_4_ evolved. Thus, to identify the genes most likely to be associated with C_4_ evolution, the sets of genes that duplicated at both origins and were retained in the descendant species were assessed for commonality. This revealed that many retained gene duplication events occurred within the same orthogroup (gene family) at both independent C_4_ origins. Furthermore, the analysis identified 21 genes that duplicated independently in parallel and were retained in the descendant species. The correspondence between the genes that duplicated in parallel at both C_4_ origins and their preferential expression in BS cell types implicates these genes in C_4_ function, and is consistent with the hypothesis that more biochemical changes happened to BS cells as compared with M cells during C_4_ evolution (Langdale 2011).

**Fig. 7:**
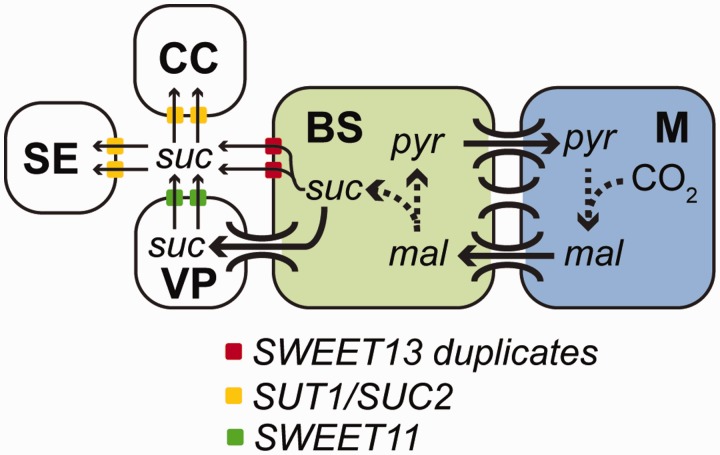
Model for localization and function of duplicated SWEET-13 genes in C_4_ photosynthesis. CC is companion cell, SE is sieve element, VP is vascular parenchyma, pyr is pyruvate, mal is malate, and suc is sucrose. Dashed arrows indicate multistep biochemical pathways. Solid arrows indicate movement of labeled compound.

Of the genes that duplicated in parallel at both C_4_ origins, several have plausible functional significance for the evolution of C_4_. Of particular note is the independent duplication of the SWEET-13 sucrose efflux transporter. In this case, although the orthologous single copy gene in *O. sativa* is expressed to a low level in mature leaves, the duplicate genes in the C_4_ lineages are each among the most highly expressed genes in the BS irrespective of whether BS was isolated as strands of vasculature (*Se.* *italic* [John et al. 2014], *So. bicolor* [presented here]) or as mechanically isolated cells (*Z. mays*; Chang et al. 2012) or laser dissected from leaf sections (*Z. mays*; Li et al. 2010). We propose that these duplicated transporters function to export sucrose out of the BS into the vasculature apoplast. This would be advantageous in C_4_ species where the number of plasmodesmata connections between BS and vascular parenchyma are reduced compared with those between the BS and M (Botha 1992; Botha and Vanbel 1992; Sowinski et al. 2007). In this model, the exported sucrose would be taken up by active proton-coupled sucrose transporter SUC2/SUT1 in the sieve elements and companion cells (fig. 7). Four lines of evidence support the role of these gene duplicates in C_4_ photosynthesis. First, the SWEET-13 gene duplicated in parallel in both independent origins of C_4_ photosynthesis in this analysis. Second, all gene duplicates are preferentially expressed in the BS of these C_4_ species. Third, expression of each of these duplicated genes is substantially higher in *Z. mays* than the related single copy ortholog in *O. sativa* (C_3_). Finally, these duplicate genes are among the highest expressed genes in the BS.

Also of note among the retained, parallel duplicated genes are the duplicates of the SQUAMOSA promoter binding-like transcription factor. The parallel duplication of this gene, combined with the neofunctionalization of one duplicate in Kranz tissue, suggest that GRMZM2G067624 may function in early vascular patterning and contribute to the patterning of Kranz anatomy in these C_4_ grasses.

It has previously been proposed that the evolution of C_4_ photosynthesis did not rely on duplication and neofunctionalization of genes (Gowik and Westhoff 2011; Williams et al. 2012; van den Bergh et al. 2014;). However, this proposal was based on the analysis of the subset of genes already known to be involved in C_4_ photosynthesis and thus there was potential for many such events to be overlooked. Here we show that there are hundreds of examples of gene duplication associated with the evolution of C_4_ photosynthesis in two separate C_4_ lineages. Moreover, we show that in at least one case a gene duplication event occurred in a gene known to be associated with C_4_ photosynthesis. This occurred in the chloroplast targeted *PPDK-rp* gene in the ancestor of *So. bicolor* and *Z. mays*. Given the expression profile of the genes descended from this duplication event and the expression profile of the single copy ortholog in *Se. italica*, it appears that this duplicate pair has undergone subfunctionalization with one duplicate preferentially expressed in BS cells and the other expressed in M cells. Thus it is likely that gene duplication and subfunctionalization played a role in the evolution of C_4_ photosynthesis in this lineage.

In agreement with previous investigations, this study revealed that several C_4_ cycle genes duplicated prior to the evolution of C_4_ photosynthesis. In all cases it appears that the same duplicate gene was co-opted for C_4_ function in both independent C_4_ lineages. Genome-wide analysis of the expression patterns of all duplicate genes that evolved on the same branch revealed that this concordance in co-option is most likely a function of expression divergence that happened prior to the diversification of the *Poaceae*. That is, the duplicate copy that is most highly expressed in leaf tissue as a result of expression divergence prior to the evolution of C_4_ is the one most likely to be co-opted for C_4_ function. Given that 74% of duplicate genes display concordant patterns of expression dominance, it will be interesting to see whether this global trend in gene co-option holds true as more C_4_ cycle genes are discovered.

Taken together this study reveals that in addition to previously documented parallelism in gene expression changes between C_3_ and C_4_ species (Gowik et al. 2011; Aubry et al. 2014), and parallelism in recruitment of *cis*-elements (Brown et al. 2011), there is significant parallelism in the evolution of new genes by gene duplication coincident with the evolution of C_4_. Furthermore, functional analysis of these newly evolved genes provides new insight into the biology of C_4_ species and suggests that duplication and neofunctionalization may have played a key role in the evolution of C_4_ photosynthesis.

## Materials and Methods

### Construction of Orthologous Gene Groups, Gene-Tree Species-Tree Reconciliation, and Filtration of Duplication Events

Protein primary transcripts were downloaded for the 41 species (supplementary file S1, Supplementary Material online) from Phytozome 10 (Goodstein et al. 2012) with genomes not under embargo in July 2014. The Brassica database version of the *Brassica rapa* genome (Cheng et al. 2011) was used in place of the Phytozome version as there were restrictions on the use of the later. Orthologous gene groups (orthogroups) were inferred using OrthoFinder (Emms and Kelly 2015). Phyldog (Boussau et al. 2013) was used to simultaneously infer the gene trees for each orthogroup and reconcile them with the species phylogeny (supplementary fig. S1*A*, Supplementary Material online). The topology of the species phylogeny for the angiosperms was obtained from the Angiosperm Phylogeny (Stevens 2001). The topology of the tree for the remaining species was obtained from Phytozome. This topology was chosen as it represents the current consensus tree for the angiosperms and is supported by a number of recent phylogenies for plants (Stevens 2001; Bremer et al. 2009; Ruhfel et al. 2014). It is worth nothing that the relationships between a small number of eudicot clades in the Phytozome tree disagree with this topology. However, these conflicting eudicot branches within the Phytozome tree are distant from the branches studied within the monocots, and thus use of the Phytozome tree would be unlikely to affect the results of the analysis. To estimate the molecular sequence branch lengths of this species phylogeny, a concatenated alignment of single copy gene orthogroups that each had representation of ≥75% of the species (*n* = 1,230) was constructed and FastTree (Price et al. 2010) was used to estimate branch lengths given the constrained species topology.

To identify the phylogenetic position of gene duplication events, the multiple sequence alignments for the amino acid sequences of each orthogroup were inferred using mafft-linsi (Katoh and Standley 2013) and the alignments subject to gene-tree to species-tree reconciliation using Phyldog and the known species tree described above. To identify retained gene duplication events the following two filtration criteria were applied: 1) A gene duplication event must have supporting evidence for its occurrence from at least two species and 2) all genes resulting from a gene duplication event must be retained in the genomes of all species that diverged after the duplication event. Thus all gene duplication events analyzed in this work are fully retained in all descendent species that evolved after the gene duplication event. The gene trees for all the gene duplication events are available from the Dryad Digital Repository, http://dx.doi.org/10.5061/dryad.17m42.

### Ontology Enrichment Analysis

GO (Ashburner et al. 2000) annotations for each orthogroup were taken from the annotations of the *A. thaliana* genes (Berardini et al. 2004) within the orthogroup. Enrichment analysis for each ontology term was performed using the hypergeometric test on the number of orthogroups with that term within a specific set (e.g., the set of orthogroups that expanded on a particular branch) given the number of orthogroups with that term in the population as a whole (e.g., the set of orthogroups containing a gene that was present on the particular branch). Correction for multiple comparisons was performed using the Benjamini and Hochberg procedure (Benjamini et al. 2001) with a false discovery rate cut-off of 0.05.

### Plant Growth and RNA Extraction

*Sorghum* *bicolor* was grown at the University of Cambridge at 28°C in a 16 h photoperiod under 400 µmol/m^2^/S photon flux density at 40% humidity as previously described (Covshoff et al. 2013). BS strands of *So. bicolor* were isolated by mechanical separation and M cell contents were collected by leaf rolling as previously described (Covshoff et al. 2013). In total, three biological replicates were collected for each cell type. Each biological replicate contained pooled RNA from the third leaf of 16 individual plants. Total RNA was extracted from the BS and M samples using the standard protocol of the mirVana micro RNA Isolation kit (Ambion).

### RNA Sequencing

RNA-seq libraries were prepared from 1 µg of total RNA (TruSeq RNA sample preparation version 2 guide; Illumina). Six libraries (comprising the three biological replicates from each cell type) were sequenced with TruSeq version 3 chemistry using one lane of the HiSeq 2000 to generate approximately 161 million 100-bp paired-end reads. The full set of raw reads from each biological replicate was deposited to EBI array express under the accession number E-MTAB-4021.

### Gene Expression Analysis

Transcriptomes from bundle sheath and mesophyll cells for *S.* *italica* (John et al. 2014), *So.* *bicolor* (described above), and *Z. mays* (Chang et al. 2012) were used to investigate the expression of duplicated genes in these specialized C_4_ cell types. In each case, paired-end reads were subject to quality trimming and adaptor filtering using Trimmomatic (Bolger et al. 2014) using the settings “LEADING:10 TRAILING:10 SLIDINGWINDOW:5:15 MINLEN:50.” The quality filtered paired-end reads were then mapped to the complete set of representative transcripts from Phytozome version 10 of the *Z. mays*, *So. bicolor*, and *Se. italica* using bowtie2 (Langmead and Salzberg 2012) and transcript abundances were estimated using Express (Roberts and Pachter 2013). All pairwise comparisons between developmental stages were made using DESeq (Anders and Huber 2010), using the default normalization method and identifying differentially expressed genes as those with a Benjamini–Hochberg corrected *P* ≤ 0.05 (Benjamini and Hochberg 1995).

## Supplementary Material

Supplementary figures S1 and S2 and files S1–S3 are available at *Molecular Biology and Evolution* online (http://www.mbe.oxfordjournals.org/).

Supplementary Data
