# Supplementary material for: Independent and Parallel Evolution of New Genes by Gene Duplication in Two Origins of C4 Photosynthesis Provides New Insight into the Mechanism of Phloem Loading in C4 Species
Source: Mol Biol Evol. 2016 Mar 24;33(7):1796–806. doi: 10.1093/molbev/msw057 (PMC4915358; doi:10.1093/molbev/msw057)
Supplement: Supplementary Data [file supp_33_7_1796__index.html]

Independent and Parallel Evolution of New Genes by Gene Duplication in Two Origins of C4 Photosynthesis Provides New Insight into the Mechanism of Phloem Loading in C4 Species — Independent and Parallel Evolution of New Genes by Gene Duplication in Two Origins of C4 Photosynthesis Provides New Insight into the Mechanism of Phloem Loading in C4 Species — Supplementary Data 

# Independent and Parallel Evolution of New Genes by Gene Duplication in Two Origins of C4 Photosynthesis Provides New Insight into the Mechanism of Phloem Loading in C4 Species

## Supplementary Data

files

- Supplementary Data - xlsx file
- Supplementary Data - pdf file
- Supplementary Data - pdf file
- Supplementary Data - xlsx file
- Supplementary Data - xlsx file
