# Supplementary figures and images for: Independent and Parallel Evolution of New Genes by Gene Duplication in Two Origins of C4 Photosynthesis Provides New Insight into the Mechanism of Phloem Loading in C4 Species
Source: Mol Biol Evol. 2016 Mar 24;33(7):1796–806. doi: 10.1093/molbev/msw057 (PMC4915358; doi:10.1093/molbev/msw057)

**A**

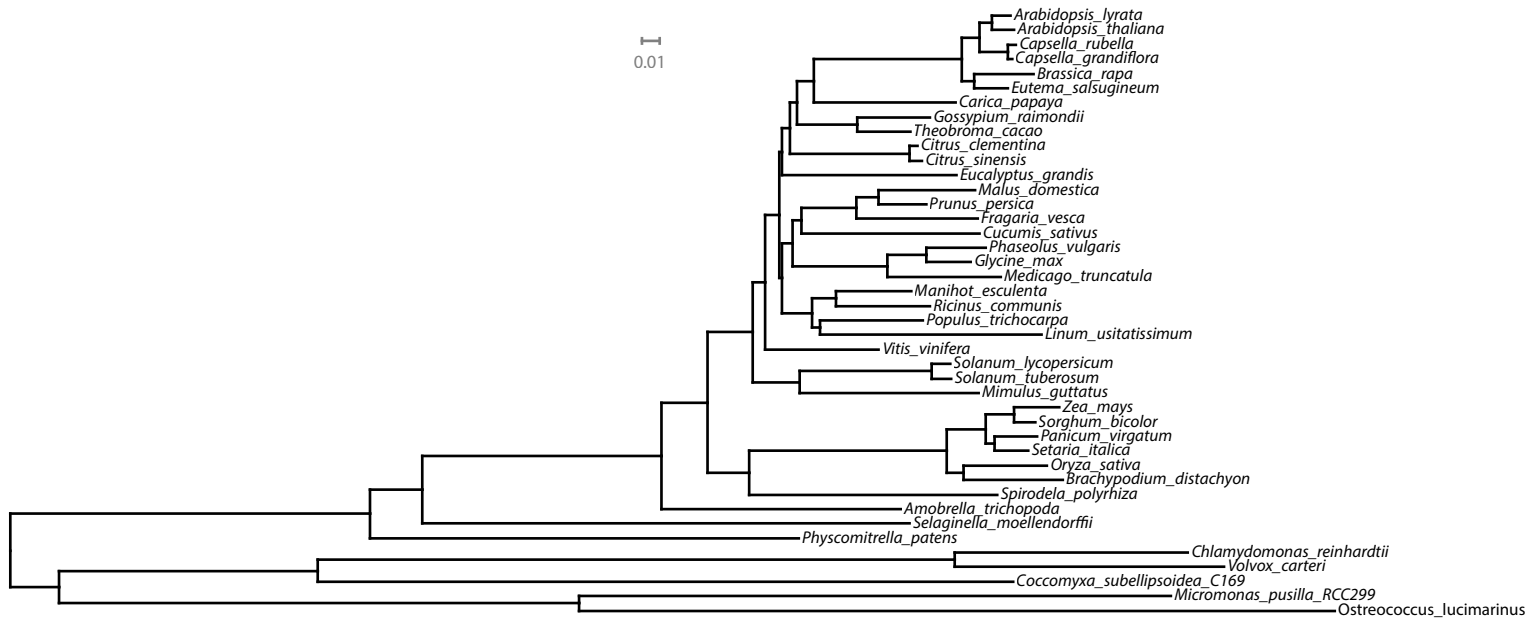

**B**

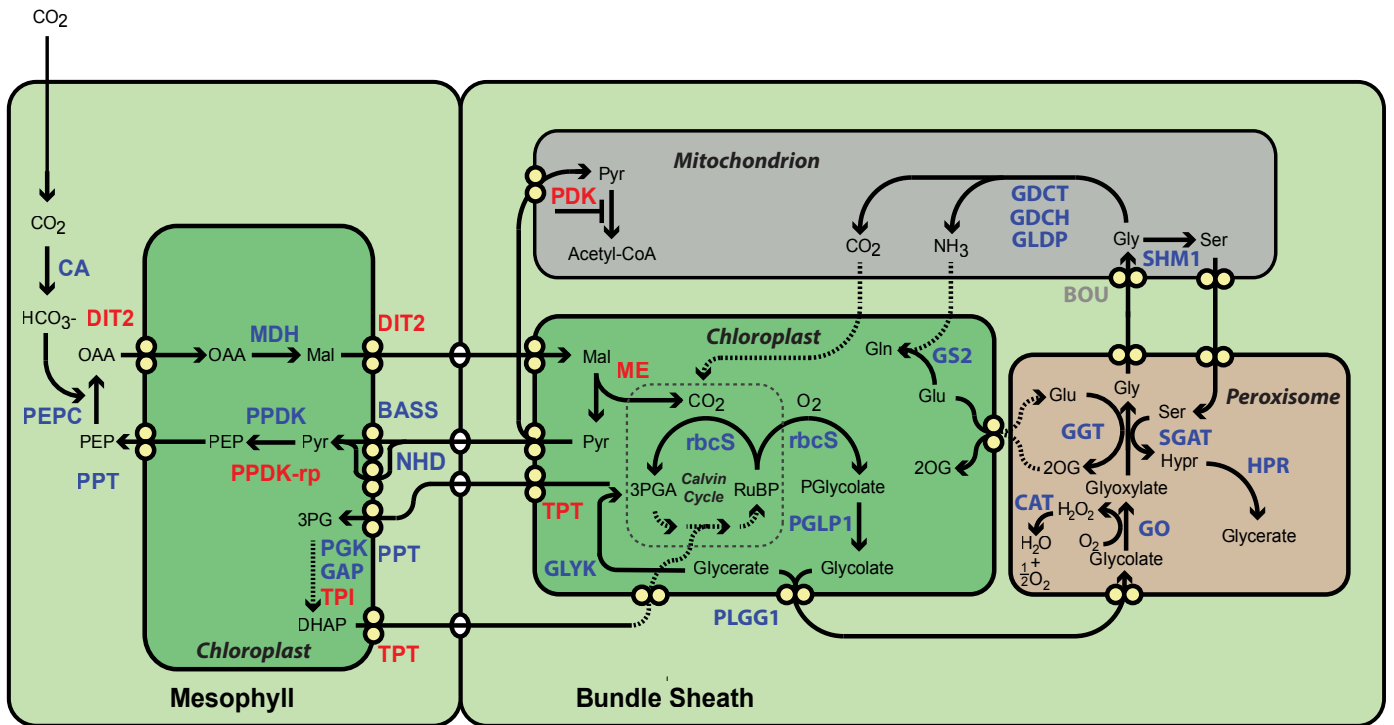

Supplement: Supplementary Data [file supp_msw057_SupplementaryFigure1.pdf]
