# Supplementary material for: Independent and Parallel Evolution of New Genes by Gene Duplication in Two Origins of C4 Photosynthesis Provides New Insight into the Mechanism of Phloem Loading in C4 Species
Source: Mol Biol Evol. 2016 Mar 24;33(7):1796–806. doi: 10.1093/molbev/msw057 (PMC4915358; doi:10.1093/molbev/msw057)

Figure 4 - figure supplement 1
Gene trees and expression profiles for the 21 parallel gene duplication events

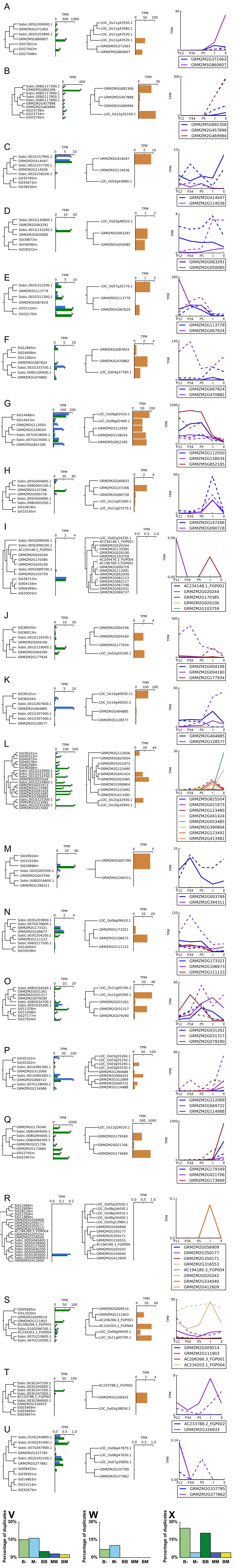

Supplement: Supplementary Data [file supp_msw057_SupplementaryFigure2.pdf]
